# Stingless bee honey, a novel source of trehalulose: a biologically active disaccharide with health benefits

**DOI:** 10.1038/s41598-020-68940-0

**Published:** 2020-07-22

**Authors:** Mary T. Fletcher, Natasha L. Hungerford, Dennis Webber, Matheus Carpinelli de Jesus, Jiali Zhang, Isobella S. J. Stone, Joanne T. Blanchfield, Norhasnida Zawawi

**Affiliations:** 1 Queensland Alliance for Agriculture and Food Innovation (QAAFI), The University of Queensland, Brisbane, QLD 4072 Australia; 2 Biosecurity Queensland, Department of Agriculture and Fisheries, Brisbane, QLD 4108 Australia; 3 School of Chemistry and Molecular Biosciences, The University of Queensland, Brisbane, QLD 4072 Australia; 4 Department of Food Science, Faculty of Food Science and Technology, Universiti Putra Malaysia, 43400 Serdang, Selangor Malaysia

**Keywords:** Mass spectrometry, Bioanalytical chemistry, Polysaccharides

## Abstract

Stingless bee (Meliponini) honey has long been considered a high-value functional food, but the perceived therapeutic value has lacked attribution to specific bioactive components. Examination of honey from five different stingless bee species across Neotropical and Indo-Australian regions has enabled for the first time the identification of the unusual disaccharide trehalulose as a major component representing between 13 and 44 g per 100 g of each of these honeys. Trehalulose is an isomer of sucrose with an unusual α-(1 → 1) glucose-fructose glycosidic linkage and known acariogenic and low glycemic index properties. NMR and UPLC-MS/MS analysis unambiguously confirmed the identity of trehalulose isolated from stingless bee honeys sourced across three continents, from *Tetragonula carbonaria* and *Tetragonula hockingsi* species in Australia*,* from *Geniotrigona thoracica* and *Heterotrigona itama* in Malaysia and from *Tetragonisca angustula* in Brazil. The previously unrecognised abundance of trehalulose in stingless bee honeys is concrete evidence that supports some of the reported health attributes of this product. This is the first identification of trehalulose as a major component within a food commodity. This study allows the exploration of the expanded use of stingless bee honey in foods and identifies a bioactive marker for authentication of this honey in associated food standards.

## Introduction

Stingless bees (Meliponini) occur in most tropical and sub-tropical regions, with over 500 species of these eusocial bees distributed across Neotropical, Afrotropical and Indo-Australian regions^1^. Like the more well-recognised *Apis mellifera* honeybees, stingless bees live in permanent colonies made up of a single queen and workers, who collect pollen and nectar to feed larvae within the colony and likewise store honey in the hive for this purpose. Honey produced by stingless bees is known by various names such as Meliponine honey, pot-honey, sugarbag honey (in Australia), and *Kelulut* honey (in Malaysia). Under these and other names, stingless bee honey has a long history of traditional indigenous use with a range of purported therapeutic properties^2^, including antidiabetic and antioxidant activity^3,4^. Various studies have been conducted of physicochemical and nutritional composition of stingless bee honey^5^, but to date few bioactive components have been identified^2^. While these studies all acknowledged that the composition of stingless bee honey is different to that of European bee honey, no rigorous identification of the major components and potential therapeutically active compounds has been reported. In addition to the importance of identifying the potential therapeutic components of stingless bee honey, the rapidly increasing consumer demand for stingless bee honey derived products has highlighted the need to produce food standards to enable establishment of authenticity and provenance of such products^6^.

**Figure 1:**
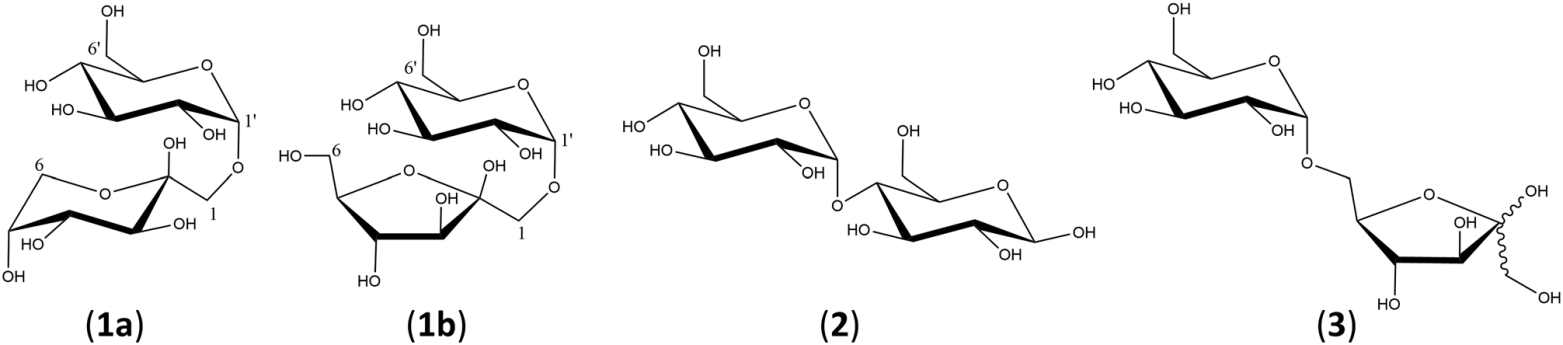
Chemical structures of trehalulose (**1**) (major, fructopyranose (**1a**) and minor, fructofuranose (**1b**) tautomers), maltose (**2**) and isomaltulose (palatinose) (**3**).

We report here for the first time that the disaccharide trehalulose (**1**) (Fig. 1) is a major component of stingless bee honeys from Malaysia, Australia and Brazil. Trehalulose is a naturally occurring isomer of sucrose, but has a much slower rate of release of monosaccharides into the bloodstream than sucrose^7,8^. This disaccharide is therefore highly beneficial in having both a low insulinemic index and low glycemic index^9^. Trehalulose is also known to be acariogenic^10,11^, and a highly active antioxidant^12^, and these properties may in no small way contribute to the reported beneficial health properties of stingless bee honey.

## Results

**Figure 2:**
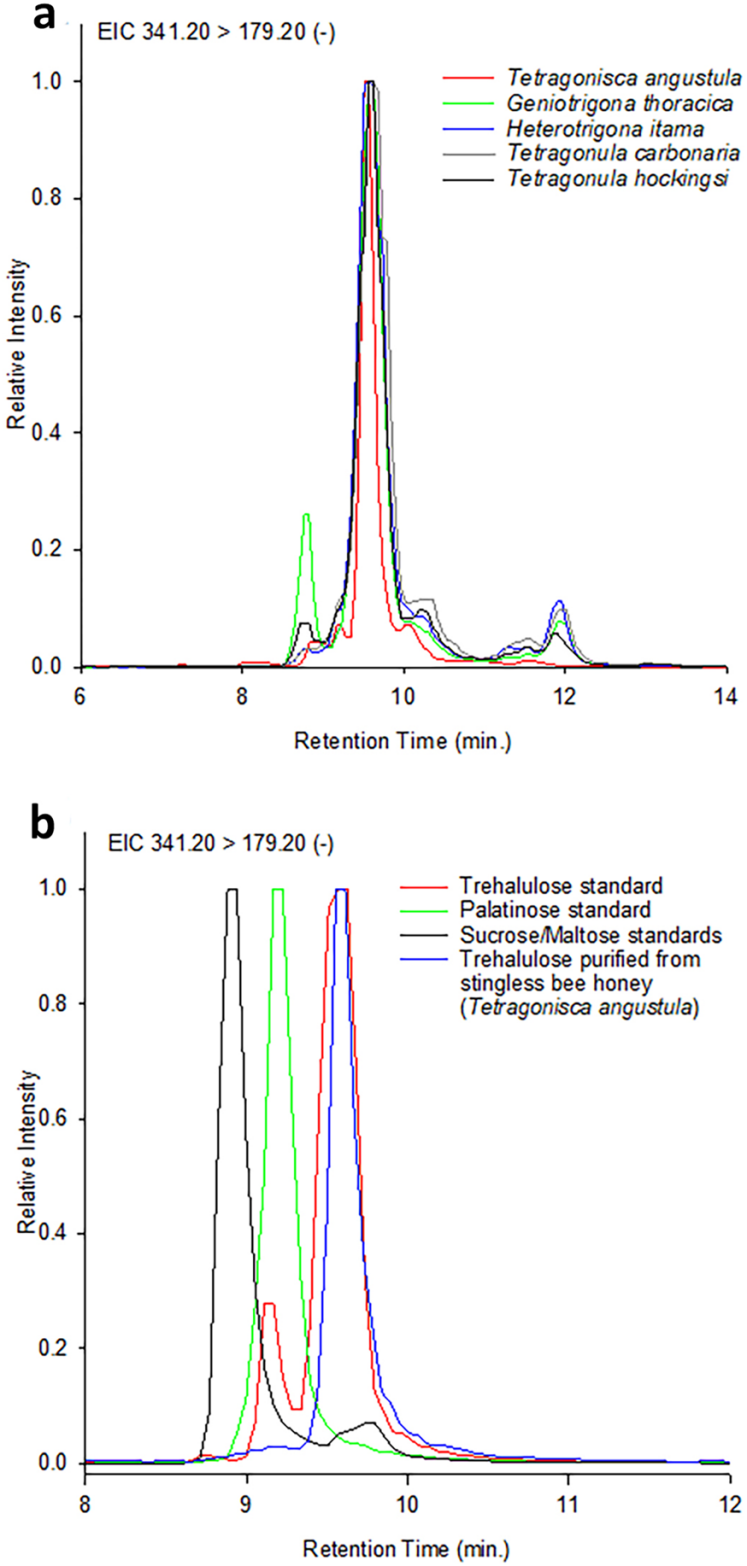
UPLC-MS/MS selected-ion chromatograms of molecular ion ([M-H]^−^) *m/z* 341.20 of: (**a**) *Tetragonula hockingsi*, *Tetragonula carbonaria, Heterotrigona itama*, *Geniotrigona thoracica* and *Tetragonisca angustula* honeys and (**b**) our isolated trehalulose, and authentic trehalulose (**1**), maltose (**2**), and isomaltulose (palatinose) (**3**).

**Figure 3:**
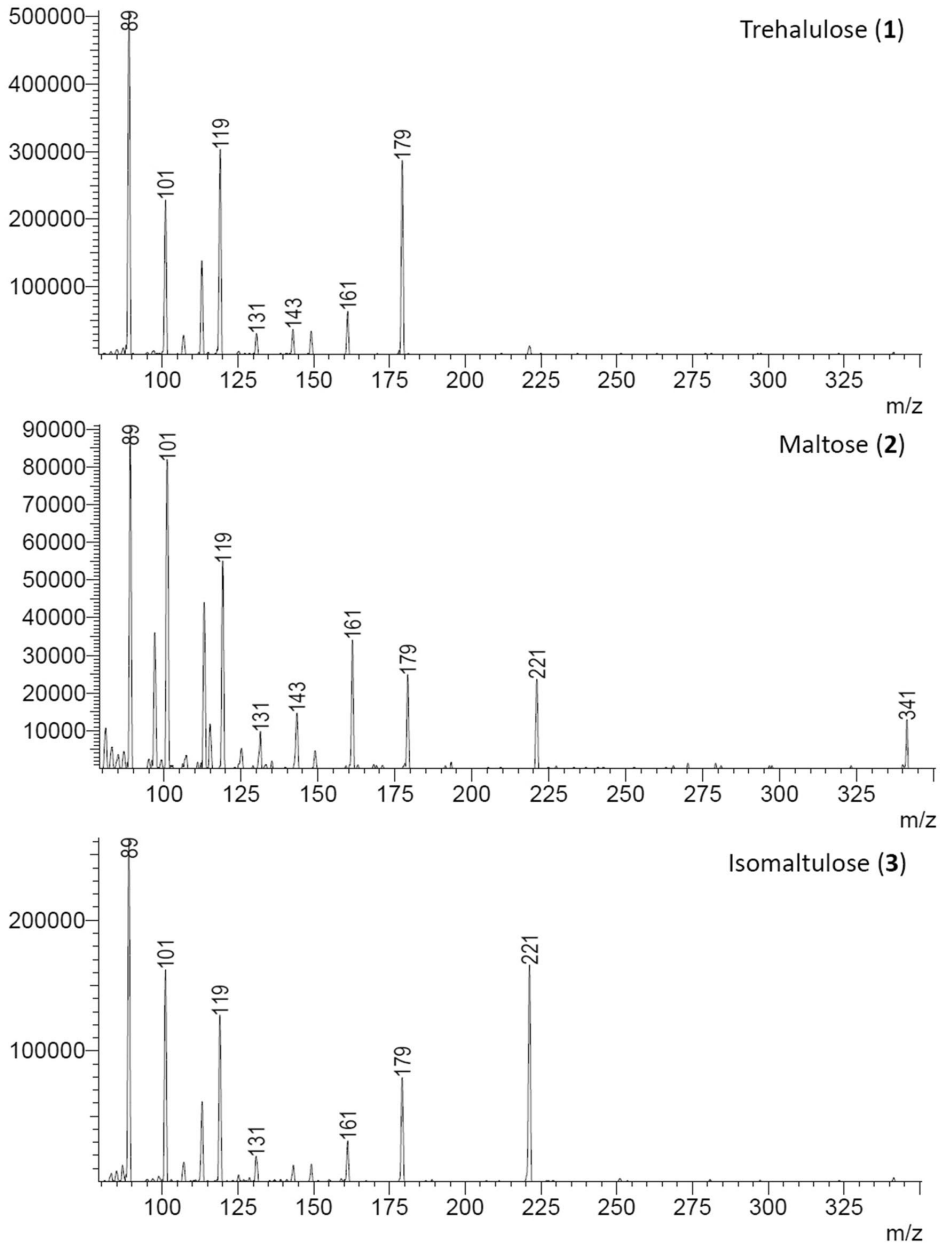
Differences in ESI–MS/MS spectra (CE 20) resulting from fragmentation of the molecular ion ([M-H]^−^) *m/z* 341 for each disaccharide (**1**–**3**) which allow these sugars to be readily distinguished.

Our UPLC-MS/MS analysis of honey from each of the five stingless bee species studied showed the presence of fructose, glucose and a single major disaccharide, the same disaccharide in each of the honeys regardless of species. From HRMS data, this major component with molecular ion ([M-H]^−^) *m/z* 341.1082 (calculated for [C_12_H_22_O_11_-H]^−^: 341.1089) was clearly a disaccharide, but did not match any of our initially available disaccharide standards. Previous analysis of stingless bee honeys have suggested that the disaccharide present was the glucose-glucose disaccharide maltose (**2**)^13–18^. However, the improved resolution and mass spectral data provided by our UPLC-MS/MS method demonstrated that the disaccharide present in all five stingless bee honeys, was in fact not maltose. This enigmatic disaccharide eluted with a slightly shorter UPLC retention time compared to a maltose standard (Fig. 2), and the mass fragmentation (MS/MS) differed from that of maltose (Fig. 3). Indeed examination of MS/MS fragmentation confirmed that it was instead a glucose-fructose disaccharide.

We have subsequently isolated the disaccharide present in samples of honey from each of the five stingless bee species by preparative HPLC and confirmed the identity of the disaccharide as trehalulose (**1**) (Fig. 1), a biologically active disaccharide that has not previously been reported in stingless bee honey.

### NMR characterisation of trehalulose

1D and 2D NMR analysis of the isolated disaccharide suggested a 1,1-linkage between glucose and fructose monosaccharides with evidence of major/minor tautomers for the fructose ring (**1a**) and (**1b**). Tentative structural assignments were compared with literature enabling the unambiguous assignment of the unknown honey disaccharide as trehalulose (Fig. 1). Trehalulose is one of only a few oligosaccharides in which the fructose ring exists predominantly in the pyranose form in solution with the 1-*O*-α-d-glucopyranosyl-β-d-fructopyranose tautomer (**1a**) being present at a higher level than 1-*O*-α-d-glucosylpyranosyl-β-d-fructofuranose tautomer (**1b**)^19^. Trehalulose was previously isolated from excrement of sweet potato whitefly *Bemisia tabaci* by Bates et al.^20^ These authors reported both ^13^C and ^1^H NMR assignments in D_2_O of three tautomers present in 20:4:1 ratio. Their NMR data were similar to incomplete details reported previously by Cookson^19^, who had isolated trehalulose from a mixture of glucose, fructose and isomaltulose produced by immobolised microbial cells, with the isolated disaccharide reported to be a 2:1 mixture of fructopyranose and fructofuranose forms by ^13^C NMR and other methods.

The 1D and 2D NMR obtained in this study confirmed that our isolated trehalulose was a mixture of both fructopyranose/fructofuranose tautomers (**1a**) and (**1b**), which were present in a 3.6:1 ratio by integration of the anomeric proton signals for the glucose rings (Table 1). The anomeric glucosyl proton signal H1′ (4.98 ppm) and fructosyl H6a (4.08 ppm) of the major fructopyranose conformer (**1a**) were irradiated in separate 1D TOCSY experiments to identify the proton signals in each monosaccharide spin system. This allowed full elucidation of the pyranose tautomer of fructose (β-d-Fru*p*) predominant in trehalulose (**1**), with assigned ^13^C and ^1^H NMR data of (**1a**) shown to be in close agreement to that previously reported by Bates et al.^20^ for this compound (Table 1) and more recently depicted by Garcia-Gonzalez et al.^21^.

**Table 1:** NMR assignments for trehalulose fructopyranose/fructofuranose tautomers (**1a**) and (**1b**).

1-*O*-α-d-glucopyranosyl-β-d-fructopyranose (1a)				1-*O*-α-d-glucosylpyranosyl-β-d-fructofuranose (1b)			
Ring	Position	^1^H NMR	^13^C NMR	Ring	Position	^1^H NMR	^13^C NMR
Glc*p*	1′	4.98 (d, 3.7)	99.32	Glc*p*	1′	5.01 (d, 3.7)	99.32
	2′	3.58 (dd, 9.9, 3.7)	72.28		2′	3.59	72.23
	3′	3.79 (dd, 9.5, 9.2)	73.81		3′	3.76	73.81
	4′	3.44 (dd, 9.8, 9.5)	70.41^a^		4′	3.44	70.29
	5′	3.73 (td, 9.8, 2.5)	72.73		5′	3.72	72.91
	6′	3.88 (dd, 12.2, 2.5)	61.31		6′	3.89	61.35
		3.80–3.76 (m)					
Fru*p*	1	3.48 (d, 10.3)	69.93	Fru*f*	1	3.56	69.29
		3.94 (br d, 10.3)					
	2	–^b^	98.64		2	–^b^	101.69
	3	3.85(dd, 10.0, 0.9)	68.70		3	4.15	77.11
	4	3.92 (dd, 10.0, 2.0)	70.34^a^		4	4.13	75.19
	5	4.02 (dd, 2.0, 1.6)	69.89		5	3.88	81.49
	6	4.08 (ABdd, 12.8, 1.0)	64.35		6	3.81	63.13
		3.72 (ABdd, 12.8, 0.9)					

NMR measured at 500 MHz for ^1^H and 125 MHz for ^13^C in D_2_O, with 1,4-dioxane (δ 67.4) for ^13^C reference.

^a^Carbon shifts may be interchanged.

^b^Quaternary carbon.

^1^H NMR resonances for the minor fructofuranose tautomer (**1b**) were partly obscured by the more dominant fructopyranose tautomer (**1a**) in the isolated trehalulose. Only the anomeric glucosyl proton doublet (H1′ 5.01 ppm, 3.7 Hz) could be clearly distinguished. Based on 2D HSQC, TOCSY and COSY experiments, all other protons and carbon shifts of the minor fructofuranose conformer (**1b**) were assigned as shown in Table 1. This full assignment extends the previous partial assignment reported by Bates et al.^20^. A third more minor tautomer with anomeric glucosyl proton doublet (H1′ 5.00 ppm, 3.7 Hz) could be seen in our trehalulose NMR spectra, but due to the low concentration and overlap with the other anomeric signals it is not possible to report full data for this compound. This is presumably the component speculated to be the α-furanose tautomer by Bates et al.^20^.

Close examination of NMR spectra demonstrated that the disaccharide isolated from each of the five stingless bees comprised > 90% trehalulose (**1**) with minor disaccharides tentatively identified as sucrose, maltulose and turanose by comparison of NMR chemical shifts with literature^22^. Isomaltulose (**3**)^22^ was not detected in these NMR spectra of trehalulose isolated from stingless bee honey.

### UPLC-MS/MS analysis of trehalulose

Literature mass spectral data for trehalulose is limited^23^, and ESI(-) MS/MS fragmentation for trehalulose (**1**) has not previously been reported. UPLC-MS/MS analysis of trehalulose isolated from each of the stingless bee species showed a single unresolved peak for (**1a**) and (**1b**) tautomers (Fig. 2). Comparison of MS/MS fragmentation with that reported for other disaccharides showed that the observed fragmentation was similar to that seen in sucrose and trehalose^24^, and consistent with the trehalulose (**1**) structure. Glycosidic bond cleavage of the molecular ion ([M-H]^−^) *m/z* 341 of trehalulose (**1**) produces a dominant *m/z* 179, with fragmentation of this ion to *m/z* 161, 143, 131, 119 and 113 (Fig. 3) being similar to that of co-occurring hexoses fructose and glucose^24^. Crucially, the fragmentation of our isolated trehalulose (**1**) differed markedly from that of maltose. Most notably, the *m/z* 221 peak corresponding to the glucosyl-glycoaldehyde anion observed in the mass spectra of maltose (**2**) and isomaltulose (**3**)^25,26^, was not observed in the 1 → 1-linked trehalulose (**1**) spectrum (Fig. 3).

The authentic standard of trehalulose obtained for confirmatory comparison exhibited a major UPLC peak with the same retention time and MS/MS fragmentation as our isolated disaccharide (**1**), but the authentic standard erroneously contained a second minor disaccharide peak which proved by UPLC-MS/MS analysis to be an isomaltulose (also known as palatinose) (**3**) contaminant. This second peak had identical retention time and MS/MS fragmentation to authentic isomaltulose (**3**) (Figs. 2, 3), including the key *m/z* 221 fragment from the loss of the tetrose l-erythrulose (120 Da)^24^. Isomaltulose (**3**) and trehalulose (**1**) are regioisomeric disaccharides which occur concomitantly in reported biological sources of these disaccharides^27^, and the presence of isomaltulose (**3**) as a 5% contaminant in the commercial trehalulose standard is understandable. Industrial trehalulose production has been achieved from sucrose using immobilised enzymes, with differing ratios of (**3**) to (**1**) produced depending on the enzyme used^28^.

It is worth noting that trehalulose (**1**) isolated by preparative HPLC from each of our stingless bee honeys contains no evidence of co-occurring isomaltulose (**3**) in either UPLC-MS/MS chromatograms (Fig. 2) or in the NMR spectra of the isolated disaccharide (**1**). This observation highlights the potential of stingless bee honey as a novel source of pure trehalulose (**1**).

UPLC-MS/MS quantitative analysis of the sugars present in the same five stingless bee honey samples employed in preparative HPLC isolations confirmed the presence of trehalulose (**1**) as a major component representing between 13 and 44 g per 100 g of each stingless bee honeys. The highest proportion of trehalulose was present in the sample of Malaysian *Geniotrigona thoracica* honey in which it represented 84% (w/w) of the total sugar content. The only other sugars present at significant levels were the monosaccharides glucose and fructose.

## Discussion

Previous analysis of sugars in honey from *Tetragonula carbonaria*^14^, *Heterotrigona itama*^15–17^, *Geniotrigona thoracica*^15,17^, and *Tetragonisca angustula*^18^ have reported that the disaccharide present was maltose, albeit accompanied with comments that the disaccharide was “not perfectly coincident with that of standard maltose”^14^. The sugar composition of honey from our fifth species *Tetragonula hockingsi* has not previously been reported. Our UPLC-MS/MS analyses have now demonstrated that the disaccharide present in each of the five honeys examined was indeed not maltose, and NMR characterisation has further shown that this disaccharide was in fact the less common trehalulose, composed of glucose and fructose joined by an α-(1 → 1) glycosidic bond (Fig. 1). Trehalulose has previously been reported as a minor sugar in some *Apis* honeys^29–31^, but this is, to our knowledge, the first documented occurrence of this unusual disaccharide as a major component of honey, and indeed the first significant natural occurrence in any food.

This novel natural occurrence of trehalulose in a food commodity is of particular interest in the food industry, due to reported health benefits associated with this disaccharide related to antidiabetic and acariogenic activity and low glycemic index^9,32^. The presence of this disaccharide as a major component in stingless bee honey is then a likely contributor to previous observations of similar biological activity attributed to these honeys. Studies have, for example, described the antidiabetic properties of *Geniotrigona thoracica* stingless bee honey, including protection against rises in fasting blood glucose levels and antioxidant properties^4^. Administration of this honey to diabetic male rats prevented increases in the level of fasting blood glucose, total cholesterols, triglyceride, and low density lipoprotein. Stingless bee honey has been shown to exhibit higher inhibition in in vitro* α*-amylase and *α*-glucosidase enzyme inhibition assays^2^. All such properties are consistent with the high level of trehalulose, rather than the previously attributed maltose, in honey from these species. Maltose has similarly been reported as a major disaccharide in honey from other stingless bee species not examined in the present study^15,33^, and it would seem likely that these reports may also reflect further misidentification of trehalulose and warrant further investigation.

This is the first isolation of trehalulose from a food source, namely stingless bee honey. Stingless bee honey represents a highly valued food with accredited biological activity, and this reported activity may in part be attributable to the previously unrecognised abundance of trehalulose in these honeys. The presence of trehalulose (**1**) as a distinguishing disaccharide in these stingless bee honeys thus provides a ready marker for authenticity, in the same way that specific marker compounds are used to authenticate high value mānuka honey^34^. Trehalulose therefore represents an ideal indicator of authenticity to be incorporated in the development of relevant stingless bee honey standards^6^. Previous attempts to develop a fast FTIR-ATR analysis of sugars in *H. itama* honey were unfortunately ill-founded in the belief that the major disaccharide present in this stingless bee honey was maltose (**2**)^16^. However, it is likely that the chemometric PLS regression analysis presented by these authors will still hold true for quantitation against trehalulose (the actual disaccharide present), presenting the ready opportunity for the development of a fast FTIR-ATR analysis method for trehalulose (**1**) in stingless bee honey. This would facilitate the development of a rapid method for the authentication of stingless bee honey products.

The long-established consumption of stingless bee honey as a therapeutic/medicinal commodity is consistent with the reported bioactivity of trehalulose as a natural sucrose isomer. Trehalulose like isomaltulose shows a reduced rate of hydrolysis in the small intestine (about one third that of sucrose)^7,8^ with application in controlling blood sugar levels for diabetes, glucose intolerance and obesity prevention. Trehalulose is 70% as sweet as sucrose and extremely water-soluble and, while not readily crystallized, has found commercial application in jellies, jams, cereal bars, juices etc^9^. Our identification of trehalulose as a major component in stingless bee honeys then provides a new, abundant and novel source for this bioactive disaccharide and opens the way to investigate the use of stingless bee honey as a food ingredient to achieve the same health benefits as attributed to pure trehalulose.

## Methods

### Stingless bee honey samples

*Tetragonula hockingsi* (syn. *Trigona hockingsi*)^35^ and *Tetragonula carbonaria* (syn. *Trigona carbonaria*)^35^ honey samples were collected from hives located in suburban backyards in Brisbane, Queensland Australia. *Geniotrigona thoracica* (syn. *Trigona thoracica*)^35^ honey was purchased from a producer in Kampung Rinching Hilir, Selangor, Malaysia. *Heterotrigona itama* (syn. *Trigona itama*)^35^ honey was purchased from a producer in Batang Kali, Selangor, Malaysia. *Tetragonisca angustula* (syn. *Trigona angustula*)^35^ honey (Brazil) was provided as a gift. All stingless bee honey samples were stored in a refrigerator (4 °C) before analysis.

### Sugar standards

Authentic trehalulose (specified by supplier > 90% purity) was purchased from Biosynth Carbosynth (Staad, Switzerland). Maltose, sucrose, glucose and fructose were purchased from Sigma Aldrich (Castle Hill, Australia), and isomaltulose from Myopure (Petersham, Australia).

### UPLC-MS/MS analysis

Honey samples (0.5 g) were dissolved in ultrapure water (50 mL) and further diluted 1:10 in water and then 1:4 in acetonitrile. Sugar standards were similarly dissolved in Millipore water and diluted sequentially in aqueous acetonitrile to provide a concentration range of glucose 1–204 µg/mL, fructose 1–195 µg/mL, sucrose 1–198 µg/mL and trehalulose 1–203 µg/mL. Analysis of sugars in individual honey samples was conducted on a Shimadzu Nexera ultra high-performance liquid chromatograph (UHPLC) coupled with a Shimadzu 8045 MS/MS detector with Lab Solutions software and a CTO-20AC column oven was operated at 35 °C. Separations were conducted on a Waters Acquity UPLC BEH Amide 1.7 µm, 2.1 × 100 mm column eluted at a flowrate of 0.2 mL/min with Mobile Phase A: 70% RO water/30% acetonitrile with 0.1% NH_4_OH and Mobile Phase B: 20% RO water/80% acetonitrile with 0.1% NH_4_OH, utilising a gradient as follows: 0–1 min, 100% B; 1–13 min, 100% B to 50% B; 13—16 min, 50% B to 100% B; 16–20 min, 100% B.

Method validation was conducted by comparing the results for calibration using external sugar standards to a standard additions method for determining sugar concentrations. For standard additions, squared linear correlation coefficients (R^2^) typically in the range of 0.98–0.99. Percentage recoveries of standard additions to five honeys at four levels for each of glucose, fructose, sucrose and trehalulose were calculated, with values for spiked samples calculated by subtraction of the endogenous, no-spike value. Recoveries for spiked samples were calculated by using the expected value and averaged 93–119% with standard deviations of 2–4% at the highest spike level, 4–15% at the intermediary spike levels and 18–40% at the lowest spike level.

The disaccharide molecular ion ([M-H]^−^) *m/z* 341.1 was fragmented at both 20 V and 8 V to examine differences in fragmentation of individual eluted disaccharides. Selected reaction monitoring (SRM) transitions of *m/z* 341.2 → 179.2 were used for quantitation of trehalulose in each of the analysed honeys with confirmation transitions of *m/z* 341.2 → 251.2 and *m/z* 341.2 → 161.2. Sucrose was quantitated using *m/z* 341.2 → 179.2, with *m/z* 341.2 → 161.2 and *m/z* 341.2 → 119.1 used for confirmation. Similarly, glucose and fructose were analysed based on SRMs of *m/z* 179.2 → 89.0 (quantitation) with *m/z* 179.2 → 101.1 and *m/z* 179.2 → 113.1 (confirmation).

High resolution mass spectral data was conducted on a Thermo Dionex Ultimate 3000 ultra high-performance liquid chromatograph (UHPLC) coupled with a Q Exactive Orbitrap high resolution accurate-mass (HRAM) spectrometry system. LC separation was carried out on a Waters Acquity UPLC BEH Amide column (100 × 2.1 mm, 1.7 μm) at 35 °C using a flowrate of 0.2 mL/min with mobile phase A: 70% RO water/30% acetonitrile with 0.1% NH_4_OH and mobile phase B: 20% RO water/80% acetonitrile with 0.1% NH_4_OH, utilising a gradient as follows: 0–1 min, 100% B; 1–15 min, 100% B to 45% B; 15–16 min, 45% B to 100% B; 16–20 min, 100% B. Analyte detection was performed by negative electrospray ionization (ESI) using full-scan ddMSMS mode using an inclusion list of 341.1089 ([M-H]^−^) and 387.1144 (M + HCOO^−^). The normalized collision energy (NCE) was set to 20%. Xcalibur (version 3.0.63, Thermo Fisher Scientific, Scoresby, Australia) was used for instrumental control and spectral inspection.

### Preparative HPLC separation of trehalulose

To enable separation of individual disaccharides, the honey samples were chromatographed on a Shimadzu HPLC-ELSD system consisting of a Shimadzu Class VP Pump LC-10AD VP/Valve FCL-10AL VP/Degasser DGU-14A with a Class VP Software/ SCL-10A VP controller, SIL-10AD VP autosampler and a CTO-10A VP column oven operated at 40 °C. Separations were performed on a Phenomenex Luna 5 µm NH_2_ 100 Å 250 × 4.6 mm column with an isocratic mobile phase comprised of 85% acetonitrile and 15% RO water at a flow rate of 2.5 mL/min. Eluted sugars were monitored with a Shimadzu ELSD- LT (Low Temperature) detector operated at 350 kPa and 45 °C. The elution flow was diverted to a collection tube when the target peak emerged and 5 mL (2 min) fractions collected before reconnection of the column eluant to the ELSD detector (to see the tail end of the peak). Collected fractions were analysed ‘as is’ by UPLC-MS/MS (as above), and a second portion freeze-dried and dissolved in D_2_O for NMR analysis.

### NMR analysis of trehalulose

The purified sugar was analysed via ^1^H nuclear magnetic resonance (NMR) analysis performed on an AV500 (500.13 MHz) Bruker Avance system using 5 mm SEI Probe. A zg45 pulse experiment at 298 K with 64 scans and a spectral width of 12.0 ppm (6,009.6 Hz) was applied. Spectra were recorded in D_2_O, with chemical shifts (δ) values recorded in ppm. Residual protonated solvent signals were used as internal standard (δ 4.80). NMR data presented as: chemical shift, multiplicity (d, doublet; t, triplet; m, multiplet; br, broad), coupling constant (*J* Hz), assignment. Multiplicities, and hence coupling constants, reported are apparent values. ^13^C NMR spectra were recorded on a Bruker Avance 500 (125.77 MHz) spectrometer with complete proton decoupling. Spectra were recorded in D_2_O with added 1% 1,4-dioxane (δ 67.40) used as reference. 2D COSY, TOCSY, HSQC, HMBC (500 MHz) spectra were used to confirm spectral assignments. Trehalulose exists in aqueous solution as a mixture of both pyranose and furanose forms (3.6:1) with both tautomers assigned as below.

1-*O*-α-d-glucopyranosyl-β-d-fructopyranose (**1a**). ^1^H NMR (500 MHz, D_2_O δ 4.80) δ 4.98 (d, *J* = 3.7, H-1′), 4.08 (ABdd, *J* = 12.8, 1.0, H-6a), 4.02 (dd, *J* = 2.0, 1.6, H-5), 3.94 (br d, *J* = 10.3, H-1b), 3.92 (dd, *J* = 10.0, 2.0, H-4), 3.88 (dd, *J* = 12.2, 2.5, H-6′a), 3.85(dd, *J* = 10.0, 0.9, H-3), 3.79 (dd, *J* = 9.5, 9.2, H-3′), 3.80–3.76 (m, H-6′b), 3.73 (td, *J* = 9.8, 2.5, H-5′), 3.72 (ABdd, *J* = 12.8, 0.9, H-6b), 3.58 (dd, *J* = 9.9, 3.7, H-2′), 3.48 (d, *J* = 10.3, H-1a), 3.44 ppm (dd, *J* = 9.8, 9.5, H-4′). ^13^C NMR (125 MHz, D_2_O, dioxane δ 67.4) δ 99.32 (C-1′), 98.64 (C-2), 73.81 (C-3′), 72.73 (C-5′), 72.28 (C-2′), 70.41 (C-4′), 70.34 (C-4), 69.93 (C-1), 69.89 (C-5), 68.70 (C-3), 64.35 (C-6), 61.31 ppm (C-6′).

1-*O*-α-d-glucosylpyranosyl-β-d-fructofuranose (**1b**). ^1^H NMR (500 MHz, D_2_O δ 4.80) δ 5.01 (d, *J* = 3.7, H-1′), 4.13 (H-4), 4.15 (H-3), 3.89 (H-6′), 3.88 (H-5), 3.81 (H-6), 3.76 (H-3′), 3.72 (H-5′), 3.68 (H-6′), 3.59 (H-2′), 3.56 ppm (H-1). ^13^C NMR (125 MHz, D_2_O, dioxane δ 67.4) δ 101.69 (C-2), 81.49 (C-5), 77.11 (C-3), 75.19 (C-4), 72.91 (C-5′), 72.23 (C-2′), 69.29 (C-1), 63.13 (C-6), 61.35 ppm (C-6′).

NMR analysis of authentic trehalulose (Biosynth Carbosynth, Switzerland) provided matching NMR data for both tautomers (**1a**) and (**1b**).

## Supplementary information

Supplementary Information.
