# Supplementary material for: Stingless bee honey, a novel source of trehalulose: a biologically active disaccharide with health benefits
Source: Sci Rep. 2020 Jul 22;10:12128. doi: 10.1038/s41598-020-68940-0 (PMC7376065; doi:10.1038/s41598-020-68940-0)
Supplement: Supplementary file 1 — Supplementary Information. [file 41598_2020_68940_MOESM1_ESM.pdf]

Stingless Bee Honey, a Novel Source of Trehalulose: A Biologically Active Disaccharide with Health Benefits

Mary T. Fletcher,[1 *] Natasha L. Hungerford,[1] Dennis Webber,[2] Matheus Carpinelli de Jesus,[3] Jiali Zhang,[1] Isobella S.J. Stone,[2,3] Joanne T. Blanchfield,[3] Norhasnida Zawawi.[4,1 *]

[1]Queensland Alliance for Agriculture and Food Innovation (QAAFI), The University of Queensland, Brisbane, QLD 4072.

[2]Biosecurity Queensland, Department of Agriculture and Fisheries, Brisbane, QLD 4108, Australia.

[3]School of Chemistry and Molecular Biosciences, The University of Queensland, Brisbane, QLD 4072.

[4]Department of Food Science, Faculty of Food Science and Technology, Universiti Putra Malaysia, 43400 Serdang, Selangor, Malaysia

*email: mary.fletcher@uq.edu.au;

norhasnida@upm.edu.my

Supporting Information

Table of Contents

Fig. S1: ^{1}H NMR spectrum of trehalulose (**1**) isolated from *Geniotrigona thoracica* in D_2O (500 MHz) 3

Fig. S2: ^{13}C NMR spectrum of trehalulose (**1**) isolated from *Geniotrigona thoracica* in D_2O (125 MHz) with 1,4-dioxane (δ67.4) for ^{13}C reference 3

Fig. S3: ^{13}C NMR DEPT45 spectrum of trehalulose (**1**) isolated from *Geniotrigona thoracica* in D_2O (125 MHz) with 1,4-dioxane (δ67.4) for ^{13}C reference 4

Fig. S4: COSY spectrum of trehalulose (**1**) in D_2O (500 MHz) 4

Fig. S5: 2D TOCSY spectrum of trehalulose (**1**) in D_2O (500 MHz) 5

Fig. S6: 2D TOCSY spectrum of trehalulose (**1**) in D_2O (500 MHz), expanded to show signals of less abundant conformer 5

Fig. S7: HSQC spectrum of trehalulose (**1**) in D_2O (500 MHz) 6

Fig. S8: HMBC spectrum of trehalulose (**1**) in D_2O (500 MHz) 6

Fig. S9: HMBC spectrum of trehalulose (**1**) in D_2O (500 MHz), expansion of H1'-C1 correlation 7

Fig. S10: HMBC spectrum of trehalulose (**1**) in D_2O (500 MHz), H1b-C1' (left), H6-C2 (Centre), H1a-C1' (right) 7

Fig. S11: 1D TOCSY spectrum of trehalulose (**1**) in **D_2O** (500 MHz), O1 2455.53, chemical shift at 5.01 ppm irradiated 8

Fig. S12: 1D TOCSY spectrum of trehalulose (**1**) in **D_2O** (500 MHz), O1 2447.27, chemical shift at 4.98 ppm irradiated 8

Fig. S13: 1D TOCSY spectrum of trehalulose (**1**) in **D_2O** (500 MHz), O1 1982.27, chemical shift at 4.08 ppm irradiated 9

Fig. S14: 1D TOCSY spectrum of trehalulose (**1**) in **D_2O** (500 MHz), O1 1961.82, chemical shift at 4.02 ppm irradiated 9

Fig. S15: 1D TOCSY spectrum of trehalulose (**1**) in **D_2O** (500 MHz), O1 1742.11, chemical shift at 3.59 ppm irradiated 10

Fig. S16: 1D TOCSY spectrum of trehalulose (**1**) in **D_2O** (500 MHz), O1 1687.46, chemical shift at 3.48 ppm irradiated 10

Fig. S17: 1D TOCSY spectrum of trehalulose (**1**) in **D_2O** (500 MHz), O1 1669.85, chemical shift at 3.44 ppm irradiated 11

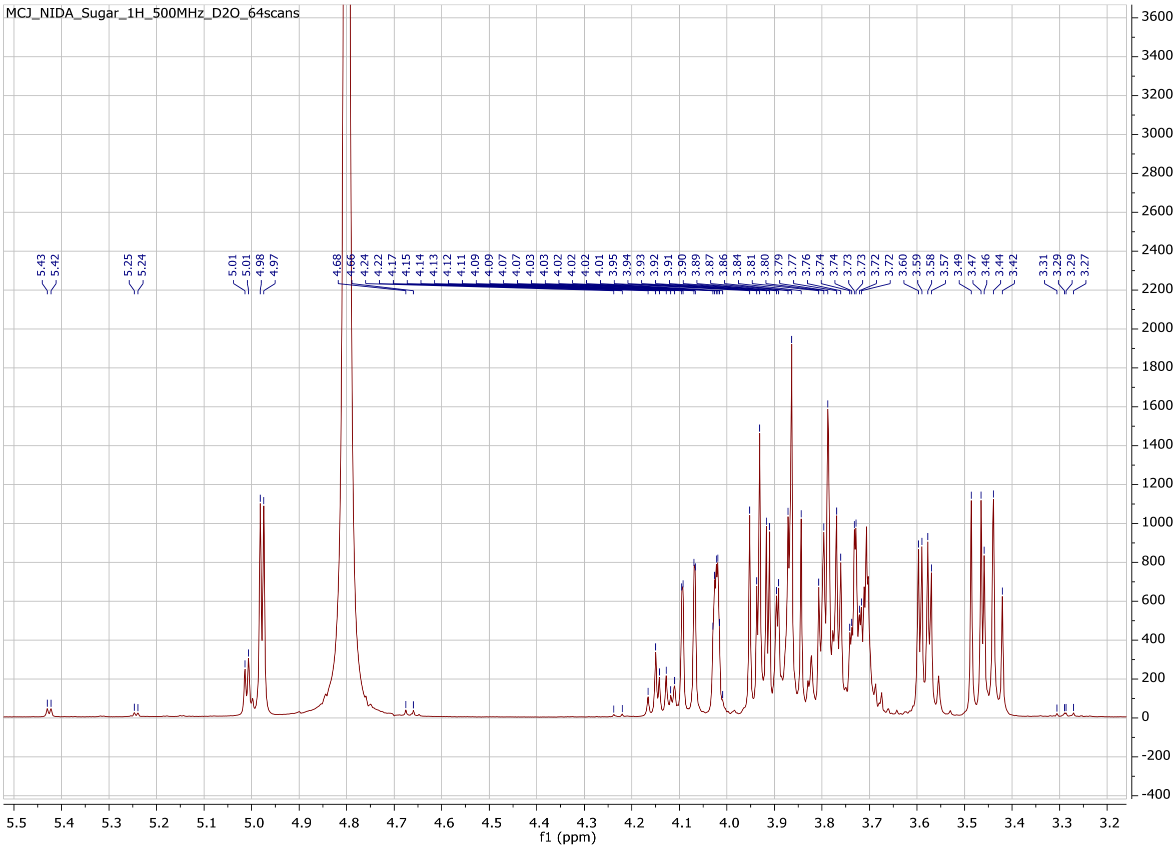

Fig. S1: ^{1}H NMR spectrum of trehalulose (**1**) isolated from *Geniotrigona thoracica* in **D_2O** (500 MHz)

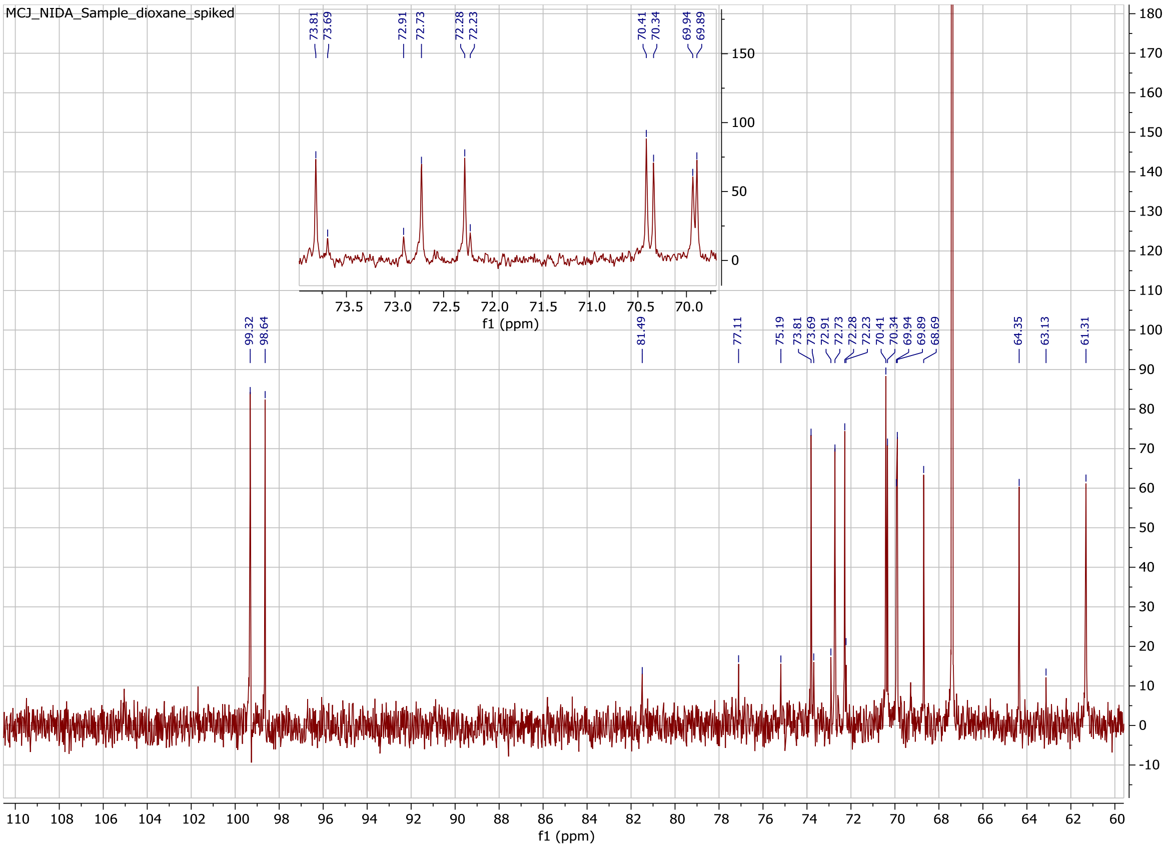

Fig. S2: ^{13}C NMR spectrum of trehalulose (**1**) isolated from *Geniotrigona thoracica* in **D_2O** (125 MHz) with 1,4-dioxane (δ67.4) for ^{13}C reference

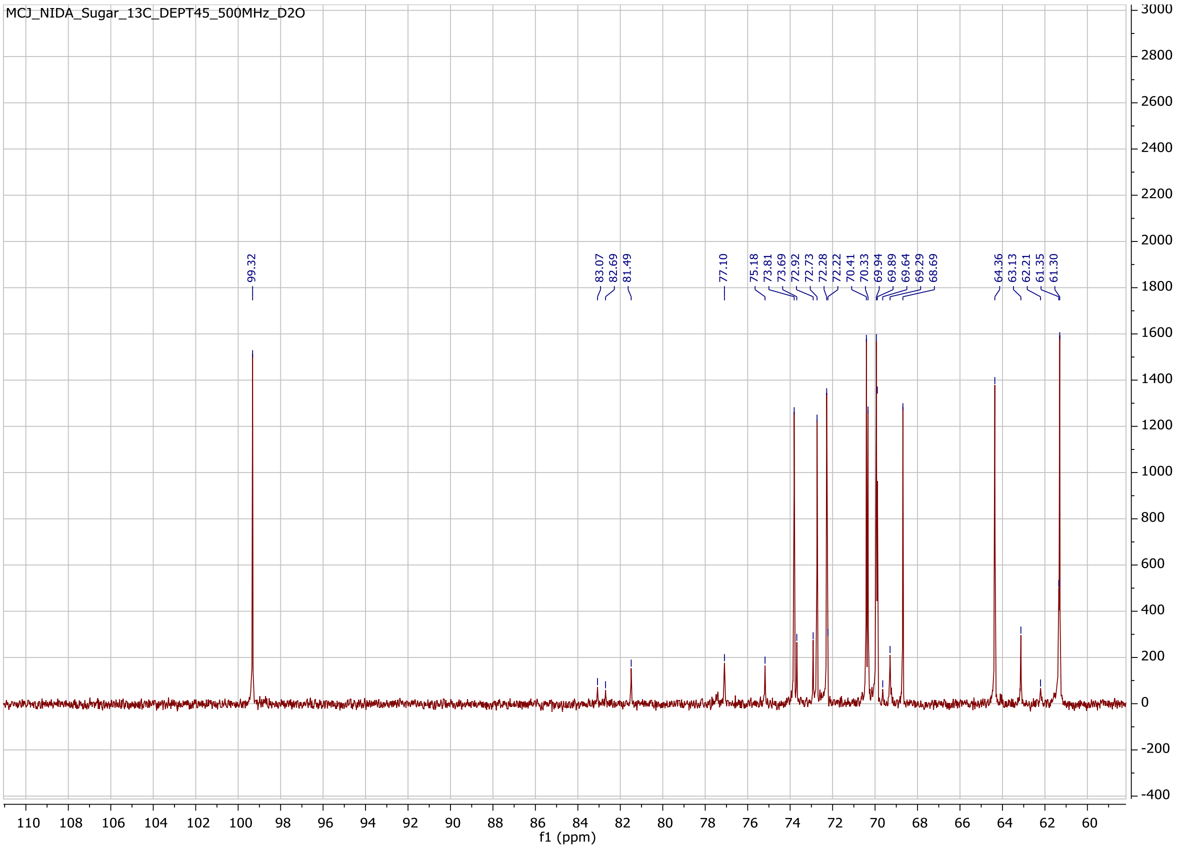

Fig. S3: ^{13}C NMR DEPT45 spectrum of trehalulose (**1**) isolated from *Geniotrigona thoracica* in **D_2O** (125 MHz) with 1,4-dioxane (δ67.4) for ^{13}C reference

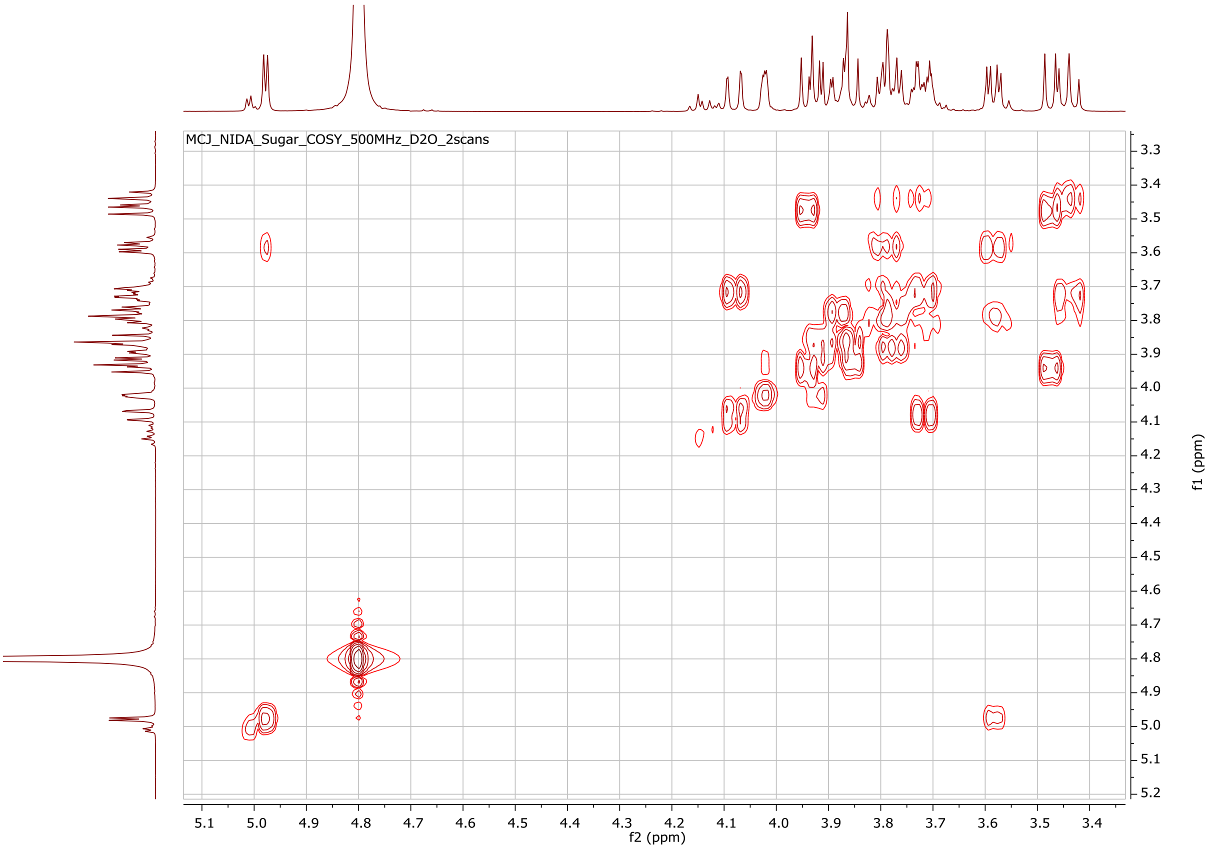

Fig. S4: COSY spectrum of trehalulose (**1**) in **D_2O** (500 MHz)

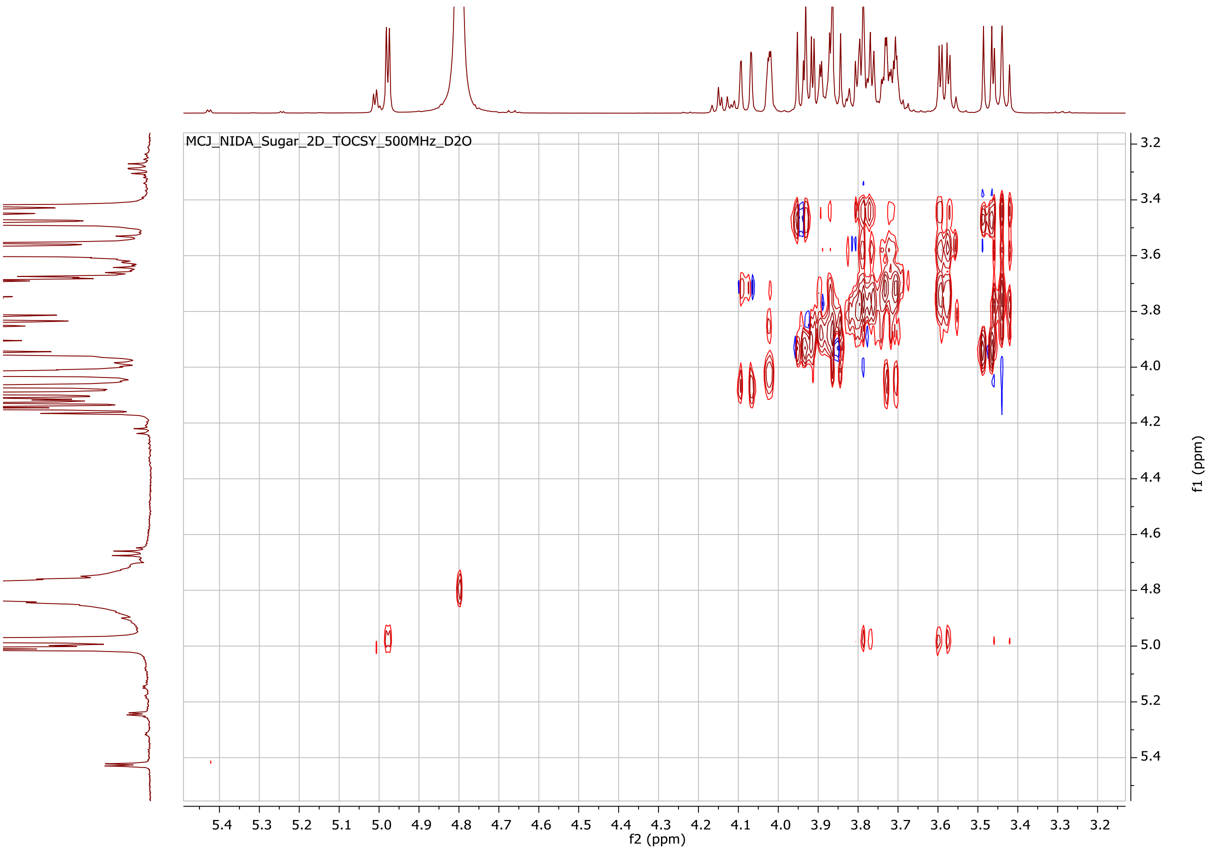

Fig. S5: 2D TOCSY spectrum of trehalulose (**1**) in **D_2O** (500 MHz)

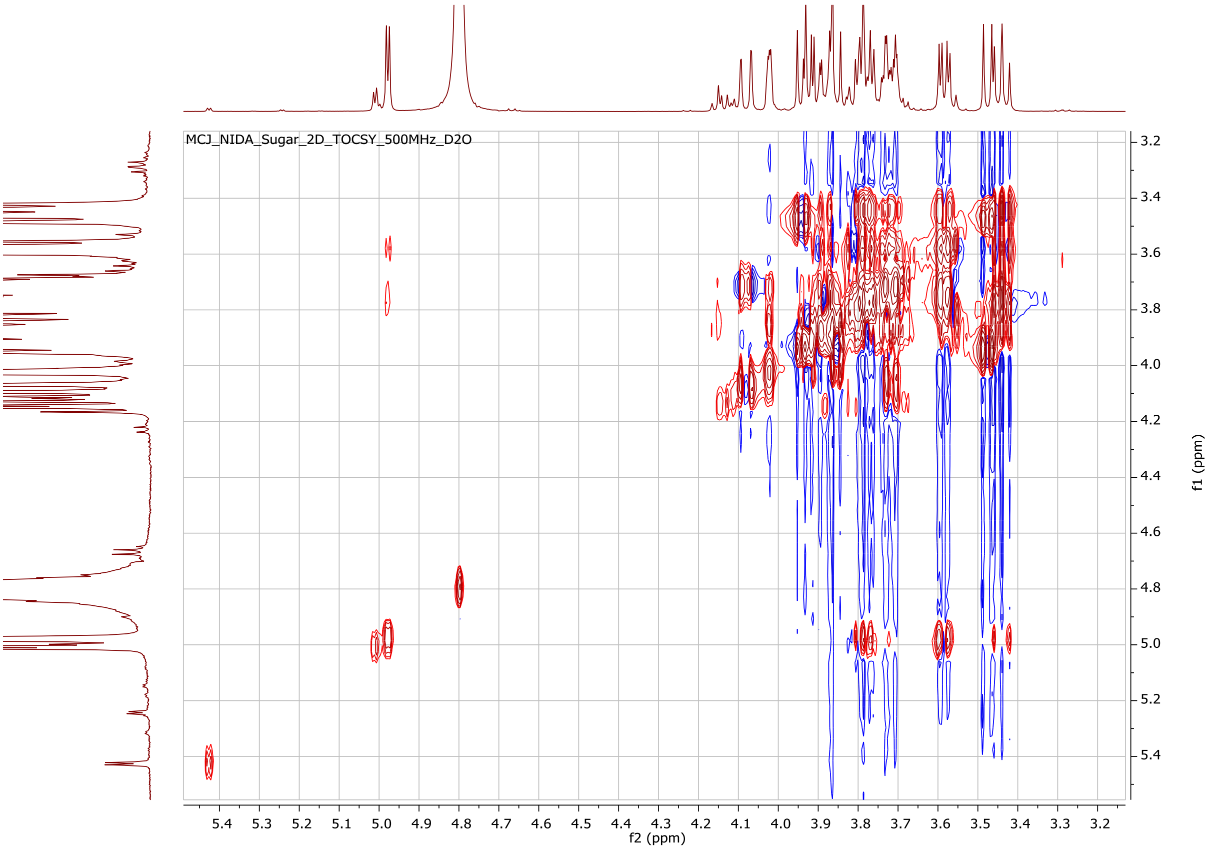

Fig. S6: 2D TOCSY spectrum of trehalulose (**1**) in **D_2O** (500 MHz), expanded to show signals of less abundant conformer

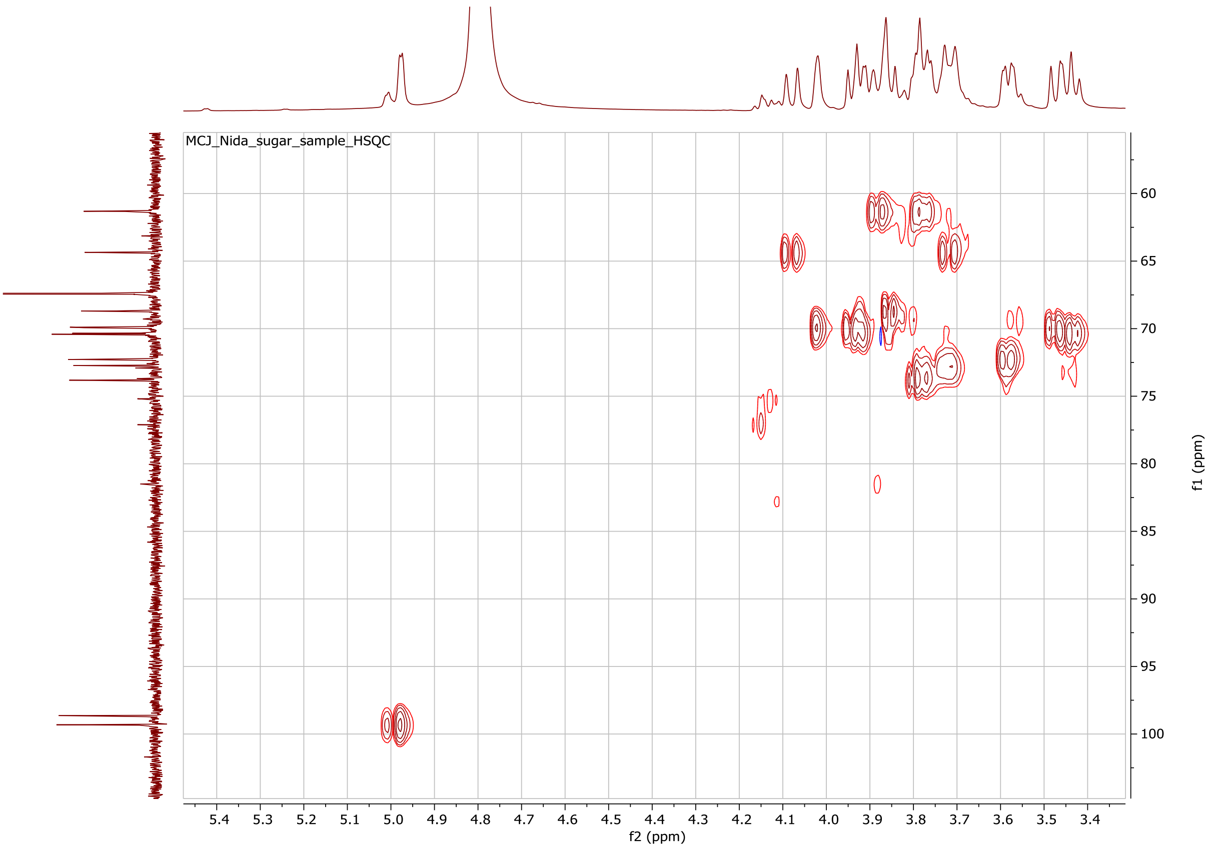

Fig. S7: HSQC spectrum of trehalulose (**1**) in **D_2O** (500 MHz)

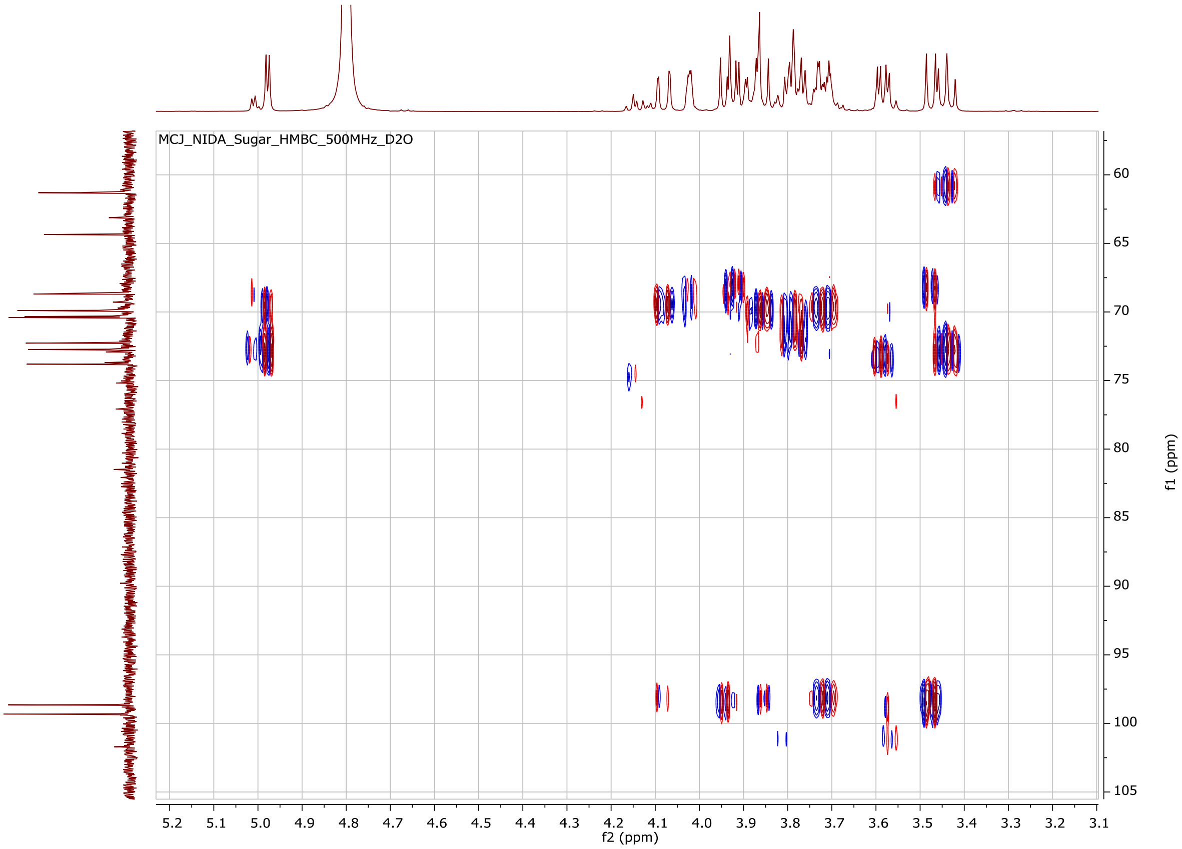

Fig. S8: HMBC spectrum of trehalulose (**1**) in **D_2O** (500 MHz)

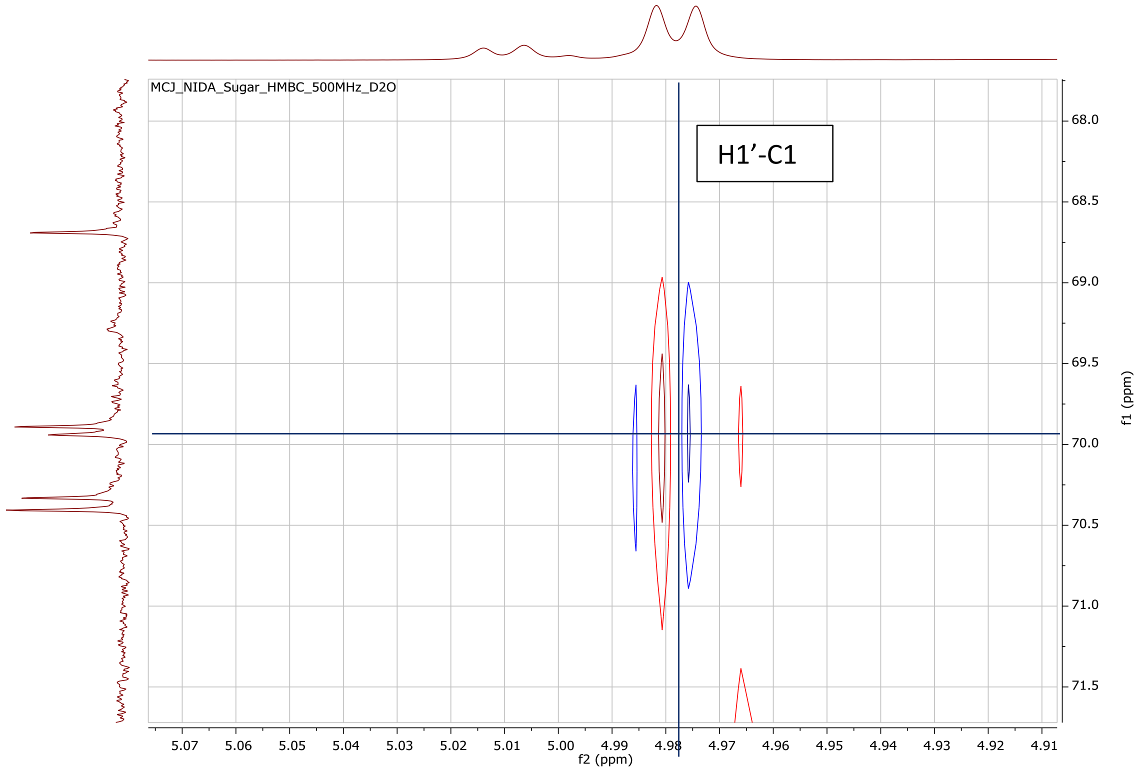

Fig. S9: HMBC spectrum of trehalulose (**1**) in **D_2O** (500 MHz), expansion of H1’-C1 correlation

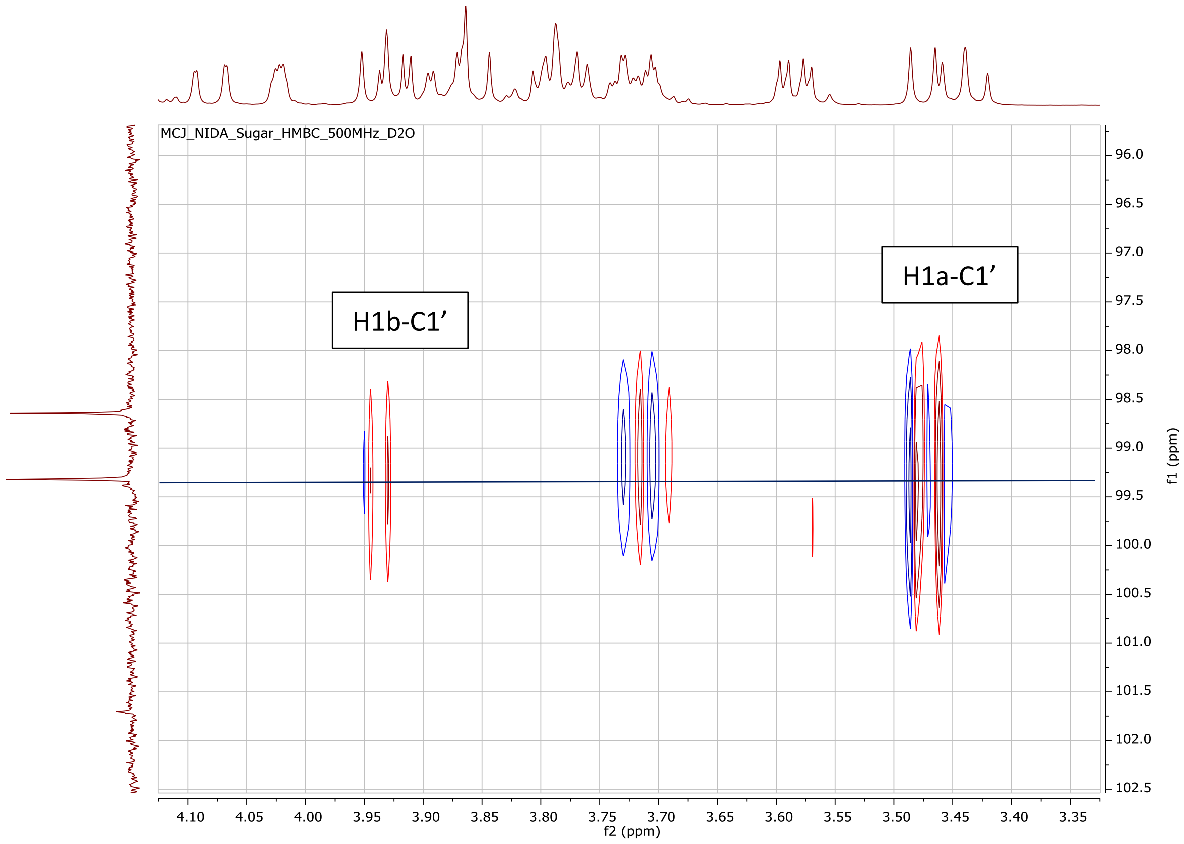

Fig. S10: HMBC spectrum of trehalulose (**1**) in **D_2O** (500 MHz), H1b-C1’ (left), H6-C2 (Centre), H1a-C1’ (right)

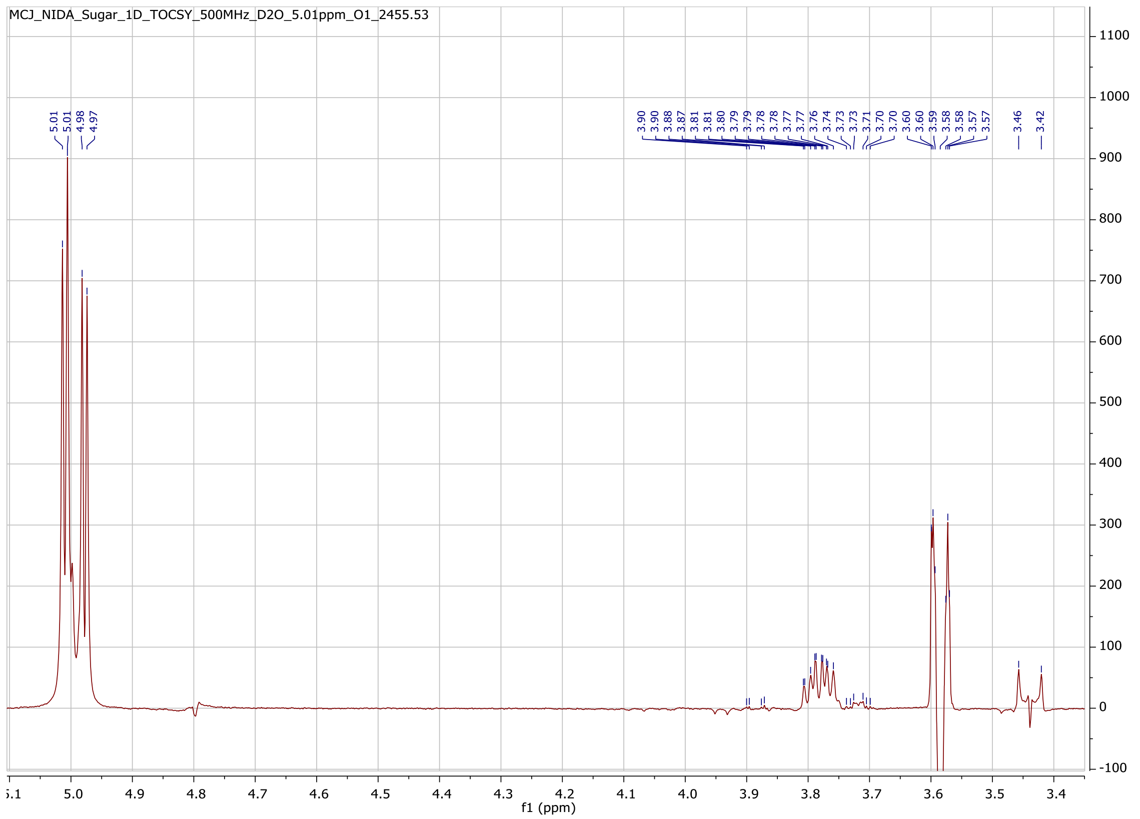

Fig. S11: 1D TOCSY spectrum of trehalulose (**1**) in $\mathbf{D_2O}$ (500 MHz), O1 2455.53, chemical shift at 5.01 ppm irradiated

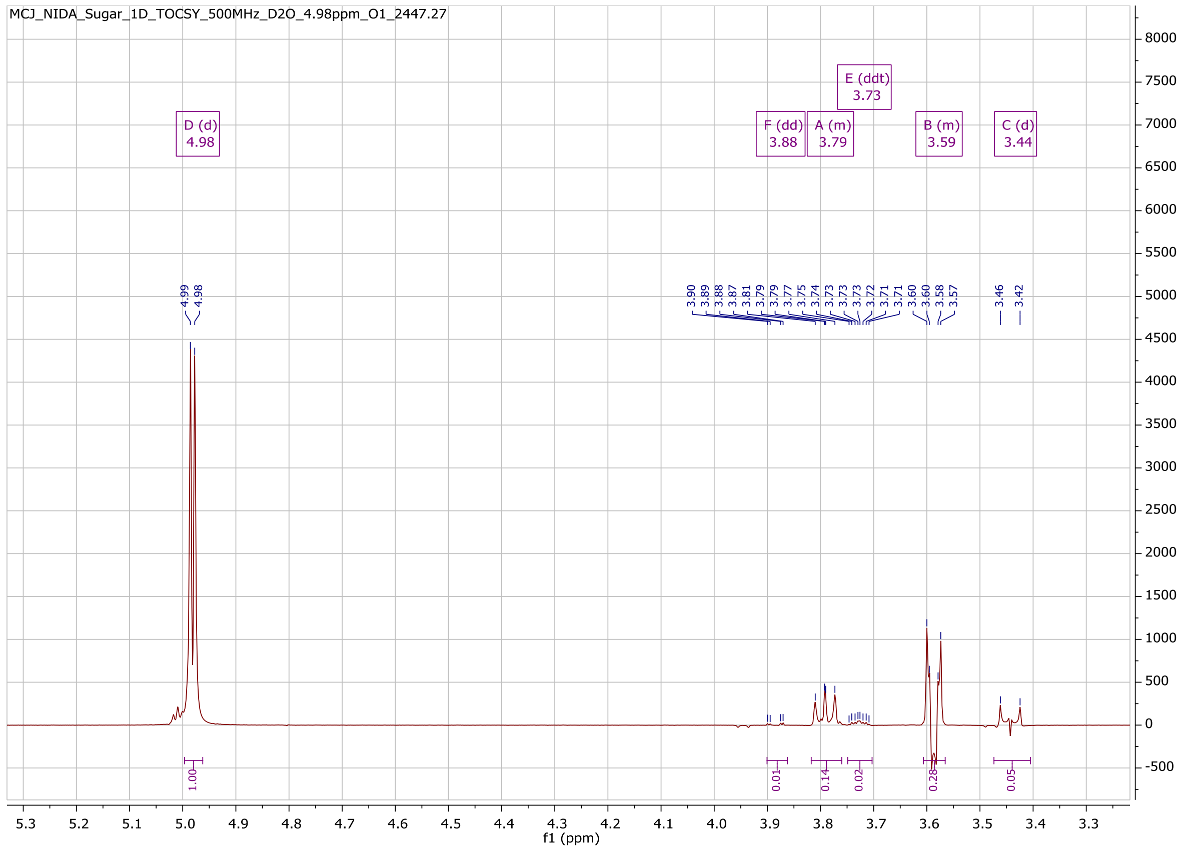

Fig. S12: 1D TOCSY spectrum of trehalulose (**1**) in $\mathbf{D_2O}$ (500 MHz), O1 2447.27, chemical shift at 4.98 ppm irradiated

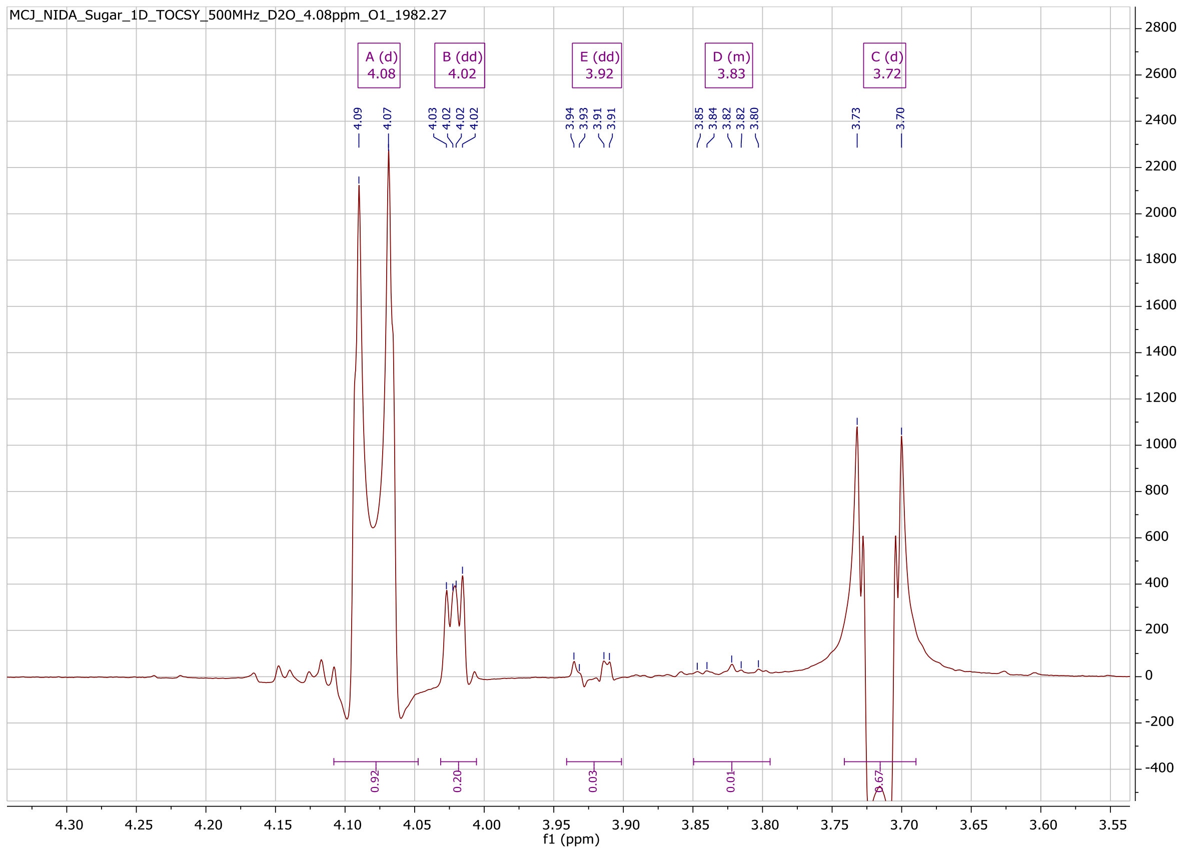

Fig. S13: 1D TOCSY spectrum of trehalulose (**1**) in **D_2O** (500 MHz), O1 1982.27, chemical shift at 4.08 ppm irradiated

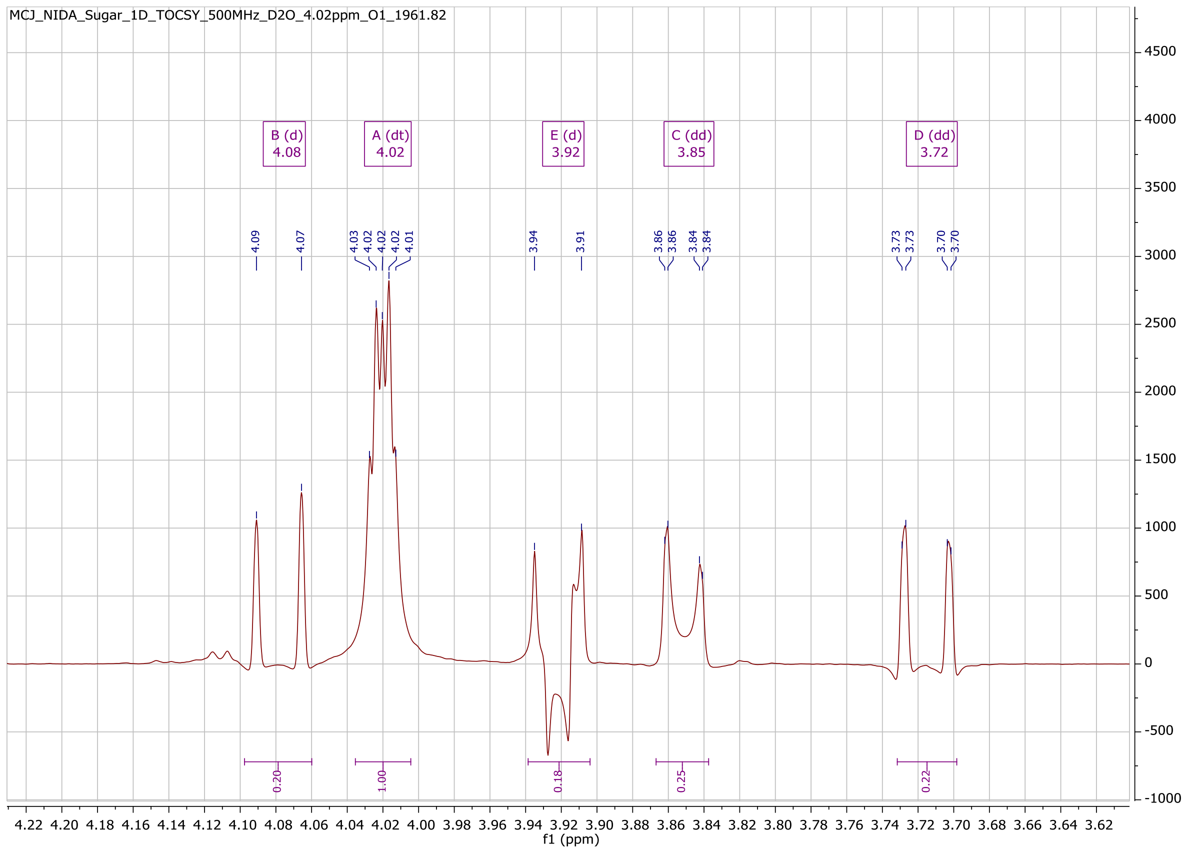

Fig. S14: 1D TOCSY spectrum of trehalulose (**1**) in **D_2O** (500 MHz), O1 1961.82, chemical shift at 4.02 ppm irradiated

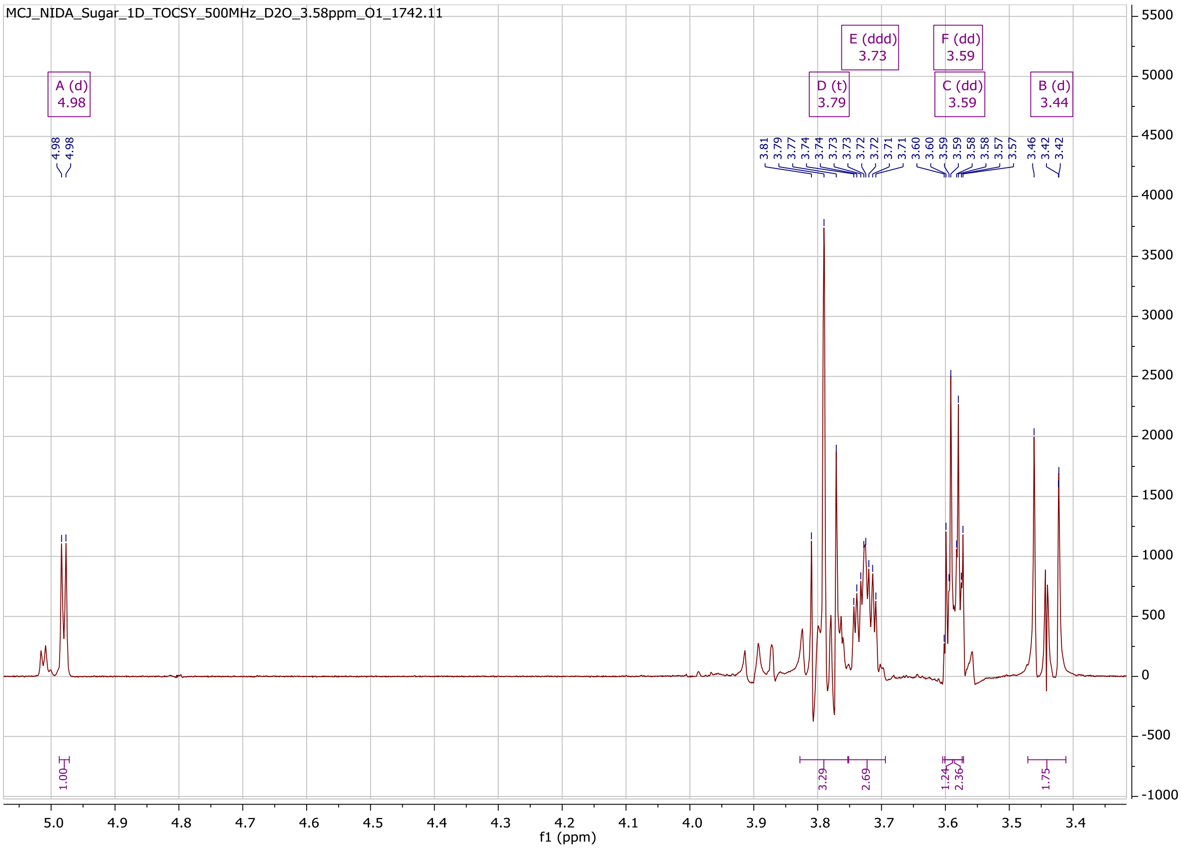

Fig. S15: 1D TOCSY spectrum of trehalulose (**1**) in $\mathbf{D_2O}$ (500 MHz), O1 1742.11, chemical shift at 3.59 ppm irradiated

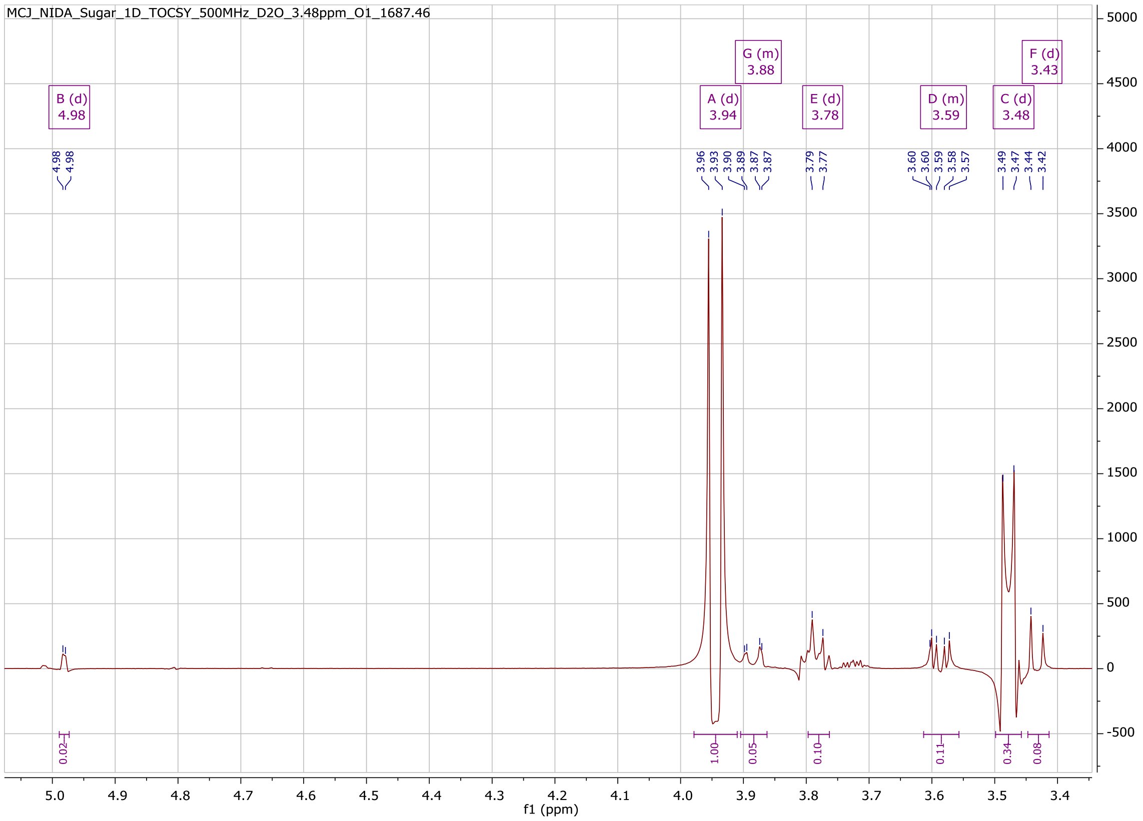

Fig. S16: 1D TOCSY spectrum of trehalulose (**1**) in $\mathbf{D_2O}$ (500 MHz), O1 1687.46, chemical shift at 3.48 ppm irradiated

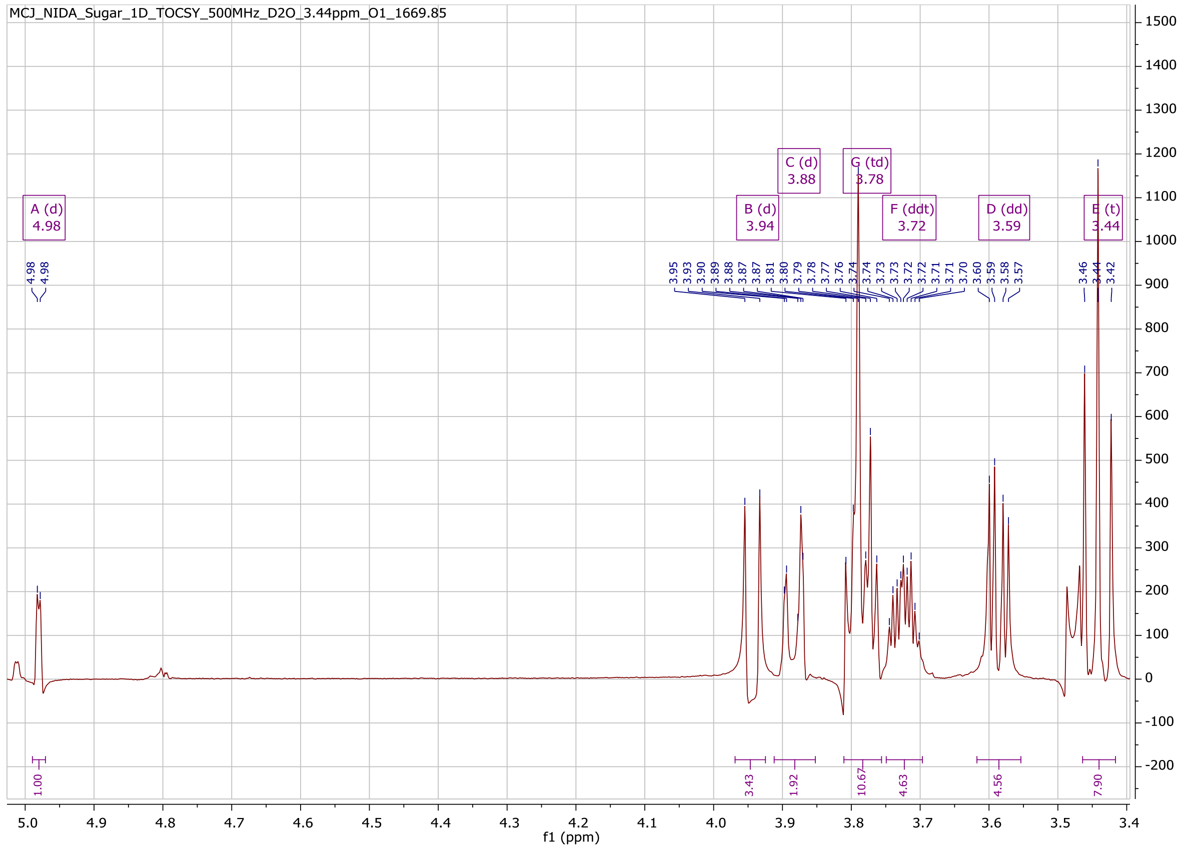

Fig. S17: 1D TOCSY spectrum of trehalulose (**1**) in **D_2O** (500 MHz), O1 1669.85, chemical shift at 3.44 ppm irradiated
